# False Impressions

**Published:** 1889-03-16

**Authors:** Caroline Fothergill

**Affiliations:** Author of "An Enthusiast," "A Voice in the Wilderness," etc.


### FALSE IMPRESSIONS.

By CAROLINE FOTHERGILL, Author of "An Enthusiast," "A Voice in the Wilderness," etc.

*(Continued from p. 368.)*

### CHAPTER XXIII.—MRS. LEDWARD CONSPIRES.

NINE o'clock had been the hour fixed by Mrs. Lester for Edith's return, and Mrs. Ledward had promised to bring her herself, disregarding Mrs. Lester's assurance that it was not in the least necessary, and that their own carriage could be sent for her. Laura listened, said very little, and quietly carried out her own scheme, as her manner was. She had the pony phaeton out, and drove Edith home with her own little capable hands, chattering gaily all the way, and rousing no suspicion in Edith's mind that she was going to crush her happiness as if it had been an inconvenient fly or moth. When they reached Queen Street she asked Edith to "run in like a good child, and see if her uncle were at home; she wished to speak to him." She used the word "uncle" on purpose, and had the satisfaction of seeing that her shaft struck home. Edith coloured, and made a little gesture of annoyance, but went indoors to do the lady's bidding.

George was at home, and, on receiving Mrs. Ledward's message, at once went out to speak to her. Laura shook hands with him, and smiled her pleasure at meeting.

"I have only a minute," she said. "I must not stay here. I am alone at home, and I want a chat with you. Come and have tea with me to-morrow afternoon."

She fancied he caught at her invitation eagerly, and she emphasised it by saying—

"Come early, I have tea at four, but come any time after after half-past two."

He promised, and she drove home with a lighter heart, thinking more of Edith than anyone else, and saying to herself—

"It will hurt her, and if she ever knows I have done it she will hate me all her life; but pain comes to us all, and it is for her good."

George came the next afternoon about three, and found Mrs. Ledward out in the garden under the trees. It was a glorious afternoon, and Laura had been out nearly all day. George liked her in the loose white muslin gown which she sometimes donned in private life, and in her big shady hat and large umbrella.

"How is it you are here alone?" he asked, as he seated himself after they had shaken hands. "You told me nothing last night, and I fancy it is seldom you and Miss Stanford are apart."

Her answer to this was,

"You see before you a perfect flawless specimen of the moral coward. I am a moral criminal, and yet to myself I can justify myself."

He begged for an explanation, and she told him of what she called "Beatrice's latest fad;" and though she reproached herself, she by no means approved of Beatrice; and she concluded with—

"I bore it as long as I could, but at last I could endure it no longer. One of the men was rude to me, and Beatrice, instead of snubbing him, laughed and encouraged him. I was very much hurt, and came home on the following Saturday. I had right on my side, but I was glad to come. I am a coward, I know."

Her cowardice did not seem to affect George deeply. He listened to her account of what Beatrice was doing with an eagerness and interest which he did not attempt to conceal, and when she had finished, he said—

"That is a splendid thing to do, and just like her. I feel quite jealous to think that she has done it, and not I. Yet, when women do take these things in hand, they beat us out and out. She is a grand woman, Mrs. Ledward," he concluded deliberately.

"You need not tell me that," she said with a little laugh. "I have known Beatrice ever since she was a baby, and although she has many faults, and some of them very grave ones, and is sometimes very difficult to live with, in spite of it all she is one of the finest women that ever lived. Although I cannot sympathise with her in what she is doing now, I know that it is a grand thing to do."

"Ay," he said, getting up, and taking a turn or two on the lawn. "It is always the same—strong light is always accompanied by strong shadow; but give me strong light all the same."

He spoke out of the fulness of his heart, and said, perhaps, more than he had intended to when he began. Laura liked to hear him; she nodded her head emphatically once or twice, and when he turned to her again he found her eyes fixed upon his face.

"This is what I wanted to come to," she said, when the pause following George's last words had lasted some little time; "but womanlike I had intended to gain my ends in a more roundabout way. I am going to ask you a very strange question, Mr. Murray, and I can't say in justification of myself that I am the only person Beatrice has to look after her, or anything of that kind. Beatrice does not need another woman to look after her; she can take care of herself better than any other woman could take care of her, so I can't pretend to be her guardian. But I am her oldest friend, and since my husband died she has always been and always will be my first thought, and latterly I have seen things which have made me feel very anxious for her. Mr. Murray, do you love Beatrice?"

There was both pathos and dignity in the manner in which Mrs. Ledward spoke and asked this question, and unconsciously she was paying by the very force of her speech a strong tribute to Beatrice's character. George had liked her from the beginning, but his admiration for her now was very deep. As soon as she had finished speaking he answered her—

"Mrs. Ledward, I love her with all the strength I possess."

Laura, who, in spite of the quiet way in which she had spoken, had felt very nervous and uncertain how what she said would be received by a man like George Murray, drew a breath of relief when his answer came. She felt no inclination to gush over this realisation of her hopes, she merely held out her hand, and said—

"Thank you."

There was a great deal more she wanted to know, but it was for George to speak now, and he did not disappoint her.

"It is my dearest wish," he said when their hands were unclasped, and they were again seated, "to win Miss Stanford for my wife, but I must candidly confess that I cannot tell from her manner if I have any chance of success."

"I could tell you, perhaps," she answered with a smile, "but I may not. You must rely on yourself alone. But I have one thing to say, one of the things, in order to say which, I asked you to come here to-day. Beatrice will be at home in a fortnight, do not think me officious if I say, speak to her at once, the very first opportunity you have. Make an opportunity if need be, I will help you; but I beg of you not to delay."

"It is my intention, especially after what you have said to-day, to speak as soon as I can; but you rather startle me with your urgency. Can you not tell me the reason of it?"

"No," was her decisive reply, "I cannot. I have observed certain things, and others have come to my knowledge, which make me think that harm is being done now, and will only be increased the longer you keep off speaking. If you speak at once, though it cannot be undone, it may be stopped; but I cannot tell you any more, and you must not ask for more. I must ask you to trust me so far."

"I trust you entirely," he said, "but your utterances are mysterious enough to make me feel very uneasy. At least tell me if she, Beatrice, is in any danger."

"No, she is in no danger," answered Laura; and then she resolutely began to talk of something else. She questioned him about his holiday in Switzerland, being wishful to get at his point of view. He told her all she wanted to know, concluding with—

"When I hear what Miss Stanford has been doing, I feel as I had shamefully wasted my time; and yet I did good, too. The change was absolutely necessary for my little niece, and I believe she enjoyed herself thoroughly."

Laura's unrestrained smile of pleasure puzzled him a little. He had no idea that she was saying to herself—

"That is just as I wished. 'Miss Lester' or even 'Edith' might have made me uneasy even now; but I shall never feel afraid of 'my little niece.'"

"She looks better," she said aloud, "and she is really charmingly pretty."

"Yes," he answered, "she is a dear child, and I am very fond of her. She is one of those people whom one has pleasure in pleasing."

No more was said about Edith. The conversation went off to other things, and after lingering until nearly dinner time, George went away.

Mrs. Ledward reviewed the interview with pleasure. It was characteristic of her that she had spoken to George rather than to Edith. To urge George to speak to Beatrice, to give him encouragement in looks and manner, even if she withheld it in words, was to place herself in a favourable light to give pleasure to increase George's regard for her. To have opened Edith's eyes would have been to incur the certainty of a very tiresome and disagreeable scene, to figure as a spiteful and meddlesome woman, to bring upon herself general ill will. She preferred the pleasanter, and therefore the easier course; for she had gauged herself truly—she was at heart a thorough coward.

She felt perfectly easy as regarded Beatrice. She had easily obtained a promise from George before he left that he would never, under any circumstances, whether Beatrice became his wife or whether she did not, betray the share she had had in arranging matters. She said if Beatrice knew what she had done she would never forgive her, even should her intervention prove to have been quite necessary. But George had promised, and she felt quite safe.

As for George himself, he walked home in high spirits. His talk with Mrs. Ledward had moved him deeply. What the mysterious "harm" at which she had hinted might be, or what things she might have observed, he did not stay to think. He was only mindful of her encouragement to speak, and her recommendation that speech should not be delayed. He began to make plans for the future, and had already arranged which rooms in Queen Street should be offered to Mrs. Ledward if she would consent to share their home, when he suddenly remembered how far off from all that he was, and pulled himself together with a short laugh.

Beatrice would be at home in a fortnight, but after hearing what he had heard this day he could no more have waited a fortnight to speak to her than he could have waited a year. "Make an opportunity to speak to her," Mrs. Ledward had said, and he determined he would. He could not wait until she came home, but he could go to Limehill and see her there. He sent no warning of his coming, but without saying a word to anyone, took an afternoon train on the day following his interview with Mrs. Ledward.

It was just tea time when he reached the house, and as he walked up the garden he saw Beatrice and Laura sitting under the trees with a couple of dogs lying at their feet. Laura had only just returned, and had not yet told Beatrice all her news. They evidently did not see him, but a sudden scorn and impatience of forms seized him. He determined to make his way to his lady at once, without the aid of any intervening servant, and he strode over a flower bed and across the lawn, and was at her side almost before she had realized who her visitor was. He had his reward. He saw how his unexpected coming moved her, how the colour rushed into her face, how she caught her breath and had some difficulty in finding words to bid him welcome.

Tears came into Laura's eyes at the pressure of his strong fingers on her little jewelled hand. She forebore to meet his eyes, and almost at once rose from her seat and began to move towards the house.

"Where are you going, Laura?" asked Beatrice promptly.

"To tell Foster to bring an extra teacup," answered Mrs. Ledward blandly, without pausing in her retreat.

"You can tell him when he comes," said Beatrice.

But Laura took no notice at all, only continuing her walk a little faster than before; and Beatrice and George were left alone.

He at once began to talk about herself, and persisted in it, in spite of her efforts to turn the conversation to other subjects. He told her a little about his own holiday and his plans for the winter, and then, suddenly and without any preface, he asked her to be his wife.

She sat silent. As we know, she had long since ceased to deny to herself that George was paramount in her thoughts. Ever since he had gained them this afternoon, after the long time in which she had not seen him, she had felt her feeling for him so intensely, as almost to deprive her of speech. At the mere sight of him, the sunshine seemed to grow brighter, the trees greener, the song of the birds more jubilant. She had fought against it long, but in vain; it mastered her at last, and when he asked her the question, the answer to which would settle her whole life, being a woman with very little circumlocution, finesse, or coquetry in her nature, she simply put her hand into his outstretched one, and said—

"With all my heart."

It is needless to say that Laura was not surprised. She was radiantly glad, and only felt a twinge of regret when George, on leaving, said—

"I must make haste to tell little Edith about this. She seemed to have some idea of it in Switzerland, and said she should like it."

*(To be continued.)*

---

That phthisis is contagious may be held as practically proved by the following fact: A young physician had been accustomed to use the same needle for hypodermic injection on himself and a consumptive patient. He died suddenly; and at the post-mortem examination there were found evidences of tubercular disease, strictly confined to the part where the needle had been used.

